# Paliperidone 3-Month Injection for Treatment of Schizophrenia: A Narrative Review

**DOI:** 10.3389/fpsyt.2021.699748

**Published:** 2021-09-21

**Authors:** Amber N. Edinoff, Prithvi K. Doppalapudi, Claudia Orellana, Caroline Ochoa, Shelby Patti, Yahya Ghaffar, Elyse M. Cornett, Aaron J. Kaye, Omar Viswanath, Ivan Urits, Adam M. Kaye, Alan D. Kaye

**Affiliations:** ^1^Department of Psychiatry and Behavioral Medicine, Louisiana State University Health Science Center Shreveport, Shreveport, LA, United States; ^2^School of Medicine, Louisiana State University Shreveport, Shreveport, LA, United States; ^3^Department of Anesthesiology, Louisiana State University Health Shreveport, Shreveport, LA, United States; ^4^Department of Anesthesiology, Medical University of South Carolina, Charleston, SC, United States; ^5^College of Medicine, University of Arizona College of Medicine-Phoenix, Phoenix, AZ, United States; ^6^Department of Anesthesiology, Creighton University School of Medicine, Omaha, NE, United States; ^7^Valley Anesthesiology and Pain Consultants – Envision Physician Services, Phoenix, AZ, United States; ^8^Southcoast Health, Southcoast Physicians Group Pain Medicine, Wareham, MA, United States; ^9^Thomas J. Long School of Pharmacy and Health Sciences, Department of Pharmacy Practice, University of the Pacific, Stockton, CA, United States

**Keywords:** paliperidone, long-acting injectable, antipsychotic, schizophrenia, Invega Trinza

## Abstract

Given the typical age onset of schizophrenia, there are tremendous economic and social impacts that extend beyond the person and their families. One critical determinant of the diseases' impact is the patient's adherence to antipsychotic drug treatment. Approved in 2015 for the treatment of schizophrenia, paliperidone palmitate (Invega Trinza, a 3-month injection, noted as PP3M) is a second-generation long-acting injectable antipsychotic medication. Among the different formulations offered for palmitate paliperidone, including the 1 and 3-month formulations, the longer duration 3-month formulation was better at preventing relapse in schizophrenic patients. To date, different formulations of palmitate paliperidone that have been studied on relapse episodes of schizophrenia include once-daily extended-release oral paliperidone (ORAL paliperidone), once-monthly paliperidone palmitate (PP1M), and once-every-3-months paliperidone palmitate (PP3M). *Post-hoc* analyses show that patients who were withdrawn from PP1M paliperidone had the least risk of relapse, followed by patients withdrawn from PP3M and patients withdrawn from ORAL paliperidone. PP3M was better at preventing relapse compared to ORAL paliperidone. The results demonstrated that 50% of patients who were withdrawn from ORAL paliperidone, PP1M, or PP3M remained relapse-free for ~2, 6, and 13 months, respectively. Compared to PP1M, PP3M is just as safe and effective and has the added advantage of increased adherence related to a longer dose interval, decreasing the risk of relapse.

## Introduction

Schizophrenia is a complex and often misunderstood mental illness that can be severely debilitating if left untreated (1). First characterized around the mid-to-late- 19th century, schizophrenia was initially described as an early form of dementia and was called “dementia praecox,” meaning “early dementia” (2). In the early 20th century, the term “schizophrenia” was used instead to distinguish mental illness from dementia and other neuropsychiatric disorders (2). Since then, technological and psychosocial advancements such as genetic testing and cognitive-behavioral therapy have drastically improved the way we understand and treat schizophrenia. However, certain aspects of the disease remain a mystery (3, 4).

Affecting ~1% of the world's population, roughly 78 million people worldwide and 2.4 million in the US, schizophrenia is one of the top 15 leading causes of disability worldwide (5). The disease often presents in early adulthood between the ages of 20 and 45, with men exhibiting symptoms in their early 20's and women in their mid-twenties to early 30's (6, 7). For affected people, all aspects of their daily lives are affected, and they have a lower life expectancy and overall quality of life (1, 8). Given the young age of onset in schizophrenia and the type of care required for patients, there are remarkable economic and social impacts that extend beyond the person and their families (9). For example, it was estimated that in 2013, ~$155 billion was spent on direct and indirect costs associated with schizophrenia, which is 2.5 times more than the approximate $62 billion spent in 2002 (8, 9). One critical determinant of the diseases' impact is the patient's adherence to antipsychotic drug treatment, which can be complicated by a number of isolated and interrelated factors such as access to care and socioeconomic status (10). Consequently, vulnerable patient populations such as low-income, minorities, and the homeless are most at risk for relapses in treatment related to lower medication adherence, resulting in uncontrolled symptoms and ultimately poorer health outcomes (9).

The use of long acting injectables (LAI) is a debated topic in the field of psychiatry. It is argued that the use of LAIs very early in the course of treatment can be very desirable as an estimated half of patients hospitalized for a first episode of psychosis discontinue their medication after being discharged (11). A study performed by Bartzokis et. al looked at the use of oral risperidone and the use of an LAI on the impact of intracortical myelination (ICM) trajectory in the first episode of schizophrenia. The authors found the ICM volume increased significantly in the LAI group and non-significantly in the oral risperidone group (12). The authors suggest that using a LAI may modify the ICM volume due to either better adherence to the medications or a different pharmacokinetic profile. Another study compared paliperidone palmitate, a 1-month LAI, with oral antipsychotic therapy. The study found that paliperidone palmitate was associated with a significant delay in time to first treatment failure vs. oral antipsychotics with overall treatment failure over 15 months being 38.8 vs. 53.7% (13). This study illustrated the real world management of schizophrenia using a 1-month LAI which demonstrated a longer time to treatment failure when compared to oral antipsychotics (13).

PP3M has shown a longer time to relapse and good safety and tolerability in many studies (14). However, its approval was met with resistance which highlighted concerns that this dosing interval would lead to less frequent doctor visits which could negatively affect the therapeutic relationship (15). This report also argued that the longer dosing interval would actually lower adherence to treatment as a whole (15). They concluded that more clinical studies should be conducted prior to the approval of PP3M to assess its safety and efficacy.

Two randomized control trials (RTCs) were used to elevate the authorization of the 3-month injection of paliperidone palmitate (PP3M). The first was by Berwaerts et al. which looked at PP3M vs. placebo for relapse prevention in schizophrenia. This study showed that the time to first relapse was significantly different in the PP3M group when compared to placebo (16). The second study was by Savitz et al. in 2016. This study was a double blind, paraellel-group multicenter phase 3 trial that was designed to test PP3M to the currently available 1-month formulation. The patients in this study were previously stabilized on the 1-month formulation. The authors found no clinically relevant differences in pharmacokinetic exposures and that PP3M was non-inferior to the 1-month with similar relapse rates (17). The authors concluded that the PP3M could offer a unique dosing option for relapse prevention in some patients. This manuscript examines the use of PP3M which shows promise in preventing relapse rates with its longer dosing interval and aims to examine the studies regarding its safety, efficacy, and clinical utility as a narrative review with the more current studies available.

## Schizophrenia Background

### Risk Factors

There is also an increased risk for future schizophrenia diagnosis after a presentation of an unspecified psychosis. According to Hensel et al., 1 in 4 persons diagnosed with unspecified psychotic disorder will receive a schizophrenia diagnosis after 2 years (18). Also, once patients have already been diagnosed with schizophrenia and have begun treatment, relapse is a possibility when patients are non-adherent to their regimens (19, 20).

### Presentation

There are three classes of findings used to diagnose schizophrenia: positive symptoms, negative symptoms, and cognitive impairment (21). Positive symptoms are also referred to as psychotic symptoms and are generally episodic in nature. They include the presence of hallucinations, delusions, or bizarre behaviors and/or beliefs. There are various classifications of hallucinations and delusions, but the common denominator is they all indicate a loss of contact with reality. According to the DSM 5, hallucinations or delusions must be present to indicate a diagnosis of schizophrenia. Negative symptoms, on the other hand, are more consistent over time and are all strongly associated with poor psychosocial functioning. These symptoms include a diminish or absence of basic emotional and behavioral states. For instance, monotonous vocal tone, immobile facial expressions, and quality of speech are examples of negative symptoms. The last set of findings in a patient with schizophrenia, the presence of cognitive impairment, are relatively intuitive. These symptoms consist of difficulties with learning, memory, attention, concentration, abstract thinking, and problem-solving (21).

## Current Treatment of Schizophrenia

Existing medications for the treatment of schizophrenia work by improving only positive symptoms such as agitation, hallucinations, delusions, and aggression (22). However, these medications are not as effective at preventing negative symptoms (23). Table 1 discusses antipsychotics.

**Table 1:** Basic mechanism of action of antipsychotics.

**Class**	**Mechanism of action**
First generation antipsychotics	D2 antagonists
Second generation	5HT2A/D2 antagonists Rapid D2 dissociation 5HT1A agonism

The only known mechanism of action of medications that are approved for the treatment of schizophrenia is the blocking of dopaminergic neurotransmission (24). This has been seen in studies looking at PET studies in patients with first break schizophrenia. Kapur et al. looked at patients prior to receiving haloperidol and 2 weeks after starting treatment. They found patients showed a wide range of D2 occupancy and the greater degree of receptor occupancy predicted clinical improvement as well as such as hyperprolactinemia and extrapyramidal side effects (25). This is consistent with the theory of a dysfunction in dopaminergic neurotransmission.

Evidence suggests that not only is the dysfunction of dopaminergic receptors responsible for the symptoms of schizophrenia, but the pathogenesis of schizophrenia also involves dysfunction of multiple signaling systems outside of the dysfunction in dopaminergic signaling (26). These systems mainly include glutamatergic, serotonergic, adrenergic, and cholinergic signaling systems (23). Therefore, new medications are being tested in Phase II and Phase III clinical trials that work on serotonin, glutamate, adrenergic, and acetylcholinergic receptors (23).

Examples of these drugs include brexpiprazole, RP-5063, and eltroprazine, which work on the malfunctioning serotonergic system (22). ADX-7114 modulates the glutamate system (26), Neboglamine modulates the adrenergic system, and ABT-126 and encenicline modulate the cholinergic system (22).

The rationale behind inhibiting the dopaminergic neurotransmission for the treatment of schizophrenia is by inhibiting the dopamine D2 receptor in the mesolimbic pathway, the psychotic symptoms of schizophrenia can be inhibited (23). On the other hand, blocking the transmission of serotonin can increase the release of dopamine in the prefrontal cortex and improve negative symptoms and cognitive impairment associated with schizophrenia (27). The agonism of the cholinergic system by nicotinic a-7 receptors is used in the control of cognitive functions associated with schizophrenia (22). Therefore, nicotinic a-7 agonists have been suggested as adjuncts to treatments that improve cognitive impairment associated with schizophrenia (23). Nicotinic agonists are used mainly for controlling the cognitive symptoms associated with schizophrenia, whereas muscarinic agonists are used to control the positive symptoms (22). The dysfunction of the glutamatergic system contributes to the development of schizophrenia in terms of negative symptoms, cognitive deficits and, possibly also positive symptoms (23). Therefore, pharmacologic modulation of this system is of great recent interest (27).

One question that can arise is, which drugs are more effective at preventing relapses and treating schizophrenia as compared to other drugs (28)? A nationwide cohort of ~30,000 patients with schizophrenia showed that clozapine and long-acting injectable antipsychotic medications prevented relapse most effectively (28).

Another important consideration is how long should the treatment be continued (29)? Relapse rates are extremely high when antipsychotic treatment is discontinued, even when the patient has suffered only a single episode of psychosis (30). Even though relapse poses serious psychological and biological consequences, there are currently no reliable predictors of relapse (29). However, treatment continued for too long leads to a poorer long-term outcome (31). Overall, whichever treatment is used for the patient, it is still best for clinicians to maintain patients on a constant low-dose, well-tolerated antipsychotic than stopping patients completely (29).

The negative symptoms associated with schizophrenia strongly affect functional outcomes; hence research and development of new drugs are important (22). However, attempts at developing anti-schizophrenia medications have had limited progress in treating negative symptoms (26). Further research is being conducted to elucidate how to improve medications to better control these symptoms (22).

## Mechanism of Action of Paliperidone

Although the exact mechanism of action of paliperidone is unclear, it is in a pathway similar to risperidone (32). That is because paliperidone is the active metabolite of risperidone. The difference between the two is the addition of a hydroxyl group in paliperidone (32). Both risperidone and paliperidone have similar binding affinities for certain receptor subtypes, there are several distinctions that are pharmacological meaningful. The differences are in the 5HT2A/D2 affinity and it is hypothesized that this difference can affect mitochondrial movement and therefore calcium homeostasis, synaptic plasticity, and neuronal firing (32). In addition to these receptors, there is differential binding to histamine, adrenergic, and cholinergic receptors. Risperidone is thought to have antagonism at the alpha 1 and alpha 2 adrenergic and H1 receiptors which may contribute to the therapeutic response as well as its adverse effects (32). Paliperidone, on the other hand, is thought to exhibit weaker affinity for the alpha 1 and alpha 2 adrenergic receptors when compared to risperidone. Other studies suggest that there is no affinity of cholinergic, muscarinic, and beta 1 and beta 2 adrenergic receptors. Paliperidone has an affinity for 5HT1D, 5HT2B, 5HT7 and D3 receptors, however, the inhibition constant values for binding to D2 and 5HT2A receptors are lower than for risperidone (32). The PP3M formulation uses NanoCrystal technology similar to its predecessor, the PP1M, but is superior in its extended sustained release capability due to an increased particle size (33).

## Paliperidone Palmitate, 3-month formulation

Paliperidone palmitate (Invega Trinza, a 3-month injection, noted as PP3M) was approved by the U.S. Food and Drug Administration (FDA) in 2015 for the treatment of schizophrenia and is a second-generation (atypical) long-acting injectable (LAI) antipsychotic medication (34). Its active ingredient is paliperidone, an atypical antipsychotic that is the metabolite of risperidone, another first-generation antipsychotics (9). At the time of FDA approval, PP3M was the only antipsychotic LAI with a 3-month interval and, in addition to treating schizophrenia, is used for schizoaffective disorder and as an adjunct to mood stabilizers or antidepressants in adults (34, 35).

### Considerations When Prescribing

Patients taking PP3M must be closely monitored for changes such as cognitive and/or motor impairment, weight, blood levels, and decreased cardiovascular function, among others (12). Atypical antipsychotics, in general, have a degree of metabolic complications such as hyperlipidemia, hyperglycemia, and QT prolongation. Additionally, certain populations are at a greater risk for complications or death while using these medications, including elderly patients with dementia-related psychosis and with renal or hepatic impairment, Parkinson's dementia, or Lewy body dementia (35, 36). The safety and effectiveness in children under 18 have not been established, and pregnant women should be advised of the potential fetal risk (36).

### Transitioning Patients to PP3M

There is a transition period before starting PP3M to safely introduce the medication to the patient (36). First, the patient must be started on a trial of oral risperidone or paliperidone to ensure tolerability and to monitor for potential side effects before being transitioned to a LAI. Once an LAI is started, patients must be stabilized on Invega Sustenna (PP1M), the 1-month version of paliperidone palmitate, for at least 4 months, with the last 2 months at the same dose (35). Only then may patients be converted to PP3M at a dose about 3.5 times higher than the last administered dose of PP1M (35, 36). PP3M is then administered in place of the next scheduled monthly injection, then every 3 months thereafter (35). The injection can be given either 1 week early or 1 week late due to scheduling issues with the patient. However, it is not approved for early injection due to treatment failure or due to the drug “wearing-off” early.

### Administration and Dosing

PP3M can only be administered by a healthcare professional and only using the thin wall needles provided in the INVEGA TRINZA® or INVEGA SUSTENNA® kits (35, 36). One dose is meant for a single intramuscular injection, and the syringe must be shaken within 5 min of injection to prevent an incomplete administration (35). If a patient misses an injection, they have up to 2.5 and 3.5 months to receive their dose (36). For missed doses of 3.5–4 months, the previously administered dose should be given immediately and then continue with the 3-month injections following this dose (36). For a missed dose of 4–9 months, they should not receive the next dose and must start a re-initiation regimen, and for missed doses that are >9 months, the patient will re-initiate treatment with PP1M before starting again with PP3M (36).

### Upcoming Advancement: 6-Month LAI

Recently, Janssen submitted a supplemental New Drug Application (sNDA) to the FDA for a 6-month formulation of Paliperidone Palmitate (PP6M) and will submit a Marketing Authorization Application extension to the European Medicines Agency (EMA) later this year (37). With an increased dosing regimen interval, the PP6M formulation hopes to offer greater flexibility and control to patients and providers for schizophrenia treatment (37). Similar to the PP3M formulation, there will be a transition period, and patients will have to be stabilized on the PP1M and/or PP3M formulations (37).

## Mortality in Studies

In 2014, 32 out of ~11,000 patients in Japan died shortly after taking Xeplion, the brand name for PP1M in that country, during post-marketing monitoring (38, 39). The reported causes of death include sudden death (most cardiac in nature), suicide, neuroleptic malignant syndrome, and other diseases such as cancer (38, 40). An analysis of these deaths, funded by Janssen Research & Development LLC., found that although there was an increased mortality reporting rate in this population, there was no significant difference in the mortality incidence rates compared to those in interventional clinical studies in Japan and in observational patient cohorts (39). Additionally, this analysis found that more than 50% of those patients were of advanced age (50+), more than 70% were at an increased risk for cardiovascular disease, and many were taking multiple antipsychotics (39). Therefore, the study concluded that the observed death rate could not be definitively attributed to Xeplion (39). However, the warning was given to not use this LAI with other antipsychotics or in a mix that could be seen as polypharmacy.

Furthermore, a meta-analysis conducted in 2016 reviewed 52 random control trials of various LAI antipsychotics (LAI-APs) to assess the safety of LAIs and found no significant difference in the incidence of death between LAI-APs and oral antipsychotics or placebo treatment groups (40). When comparing the pooled LAI-APs group to the placebo group within the first 13 weeks of treatment, there was a downward trend in the mortality rate, but the authors noted this trend with caution due to a small sampling size (40). People with schizophrenia have an increased risk of cardiovascular disease (CDV), and a significant number of deaths result from CDV (41). This increased risk has been long established, but numerous genetic, environmental, and pharmacological factors complicate this relationship (42). In view of this and the study's results, it can be concluded that there is no significant increased risk in the mortality rate while on LAI-APs (40).

## Pharmacokinetics/ Pharmacodynamics

Following injection, PP3M dissolves slowly due to it being water-insoluble (36). After dissolving, paliperidone palmitate is hydrolyzed to paliperidone and absorbed into the bloodstream. The FDA reports that once in the bloodstream, the drug reaches maximum plasma concentrations after a median of 30–33 days (36). The distribution of the drug once in circulation varies depending on injection site. Deltoid muscle injections showed an 11–12% higher maximum serum concentration on average than gluteal muscle injections (36). In the same way, deltoid muscle injections had a mean steady-state ratio of 1.7, with gluteal muscle injections having a mean peak-to-trough ratio of 1.6 (36). Overall, the drug has shown to have a volume of distribution of about 1,960 L.

Similarly, half-life has proved to differ based on injection site. The FDA reports a median half-life of 84–95 days with deltoid administration and 118–139 days with gluteal administration (36). One possible explanation for this extended half-life is that the drug is not greatly metabolized by the liver. If the provider notices that it seems to be “wearing-off” early, they should consider gluteal over deltoid injections for the previously stated reasons. The FDA demonstrated that 59% of an oral immediate-release paliperidone is excreted unchanged a week after administration, insinuating that there are no liver isozymes, largely impacting the metabolism of the drug (36).

## Difference Between PP1M and PP3M

PP1M (Invega Sustenna) and PP3M (Invega Trinza) are both intramuscular injections of paliperidone palmitate used for long-lasting treatment of Schizophrenia. PP3M is a 3-month injection, meaning it is administered once every 3 months, while PP1M is administered once a month (11). PP3M is indicated for treatment only after patients have been treated with PP1M for 4 months, and it has shown to be effective and tolerated (43). The key advantage of either paliperidone palmitate injection is that they assist with non-compliance. Inconsistency with or absence of maintenance therapy is a key factor related to relapse in schizophrenia patients (19). Furthermore, up to 80% of patients with schizophrenia do not adhere to medication regimens. This can lead to hospitalization, episodes of psychotic behavior, and overall negative burdens on not only patients but also their families and society (20). Having long-lasting treatment options available helps to alleviate some of the non-compliant aspects of the patient population (20). PP3M has the added convenience of only being required every third month as opposed to monthly. Even when comparing the pharmacokinetics of the two, the exposure for a 3.5-fold higher dose of PP3M is similar to the corresponding dose of PP1M (36).

## Clinical Studies: Safety and Efficacy

Clinical studies have been conducted recently to highlight the safety and efficacy of different medications used in the treatment of schizophrenia, especially in scenarios of non-adherence and lack of access (44). Of the many medications that have been studied, the most extensively studied medication is palmitate paliperidone, as it can be given in the injectable form and can be given for a long period of time (33). One such study compared the medication aripiprazole once-monthly 400 mg and paliperidone palmitate once-monthly on the Heinrichs–Carpenter Quality-of-Life Scale (QLS) (45). QLS is an accepted health-related quality of life measurement in schizophrenic patients (45). This study, conducted over a period of 28 weeks, showed that patients who had taken aripiprazole 400 mg had significant improvements in the metrics measured in QLS as compared to schizophrenic patients who were administered paliperidone palmitate (45).

Another similar study conducted demonstrated that among the different formulations offered for palmitate paliperidone, including the 3-month formulation and 1-month formulation, the 3-month formulation was better at preventing relapse in schizophrenic patients (44). Furthermore, another study conducted to compare the prevention in relapse in schizophrenic patients offered palmitate paliperidone 3-month formulation compared to placebo treatment showed that palmitate paliperidone 3-month formulation was better at preventing relapse (46). Moreover, schizophrenic patients who had been administered PP3M had fewer reported hospitalizations for psychiatric and social reasons as compared to patients who were given placebo (47).

It is interesting to note that the efficacy of palmitate paliperidone 3-month formulation was noted not only in the American population but was also noted in the Latin American population. A study conducted showed that Latin American patients administered palmitate paliperidone showed no new adverse effects as compared to American patients and patients from all over the world (17).

Other studies done on palmitate paliperidone are concerned with the half-life of the drug and relapse (48). The major concern is if different formulations of the drug with different half-lives affect schizophrenic patients who have discontinued the medication (49). The different formulations of palmitate paliperidone that have been studied to examine the effect of half-lives on relapse episodes of schizophrenia include once-daily extended-release oral paliperidone (ORAL paliperidone), once-monthly paliperidone palmitate (PP1M), and once-every-3-months paliperidone palmitate (PP3M) (48). *Post-hoc* analyses have shown that patients who were withdrawn from PP1M paliperidone had the least risk of relapse, followed by patients withdrawn from PP3M and patients withdrawn from ORAL paliperidone (48). PP3M was better at preventing relapse compared to ORAL paliperidone. The results demonstrated that 50% of patients who were withdrawn from ORAL paliperidone, PP1M, or PP3M remained relapse-free for ~2, 6, and 13 months, respectively (48).

Studies that have assessed and compared the pharmacokinetics, safety, and tolerability of PP3M with PP1M have shown that the overall difference between the two in these parameters is negligible (33, 50). Studies have also interestingly shown no difference in injection site pain between the two formulations regardless of dosage difference and volume difference (51). The studies also clarify that giving these medications once every 3 months is the best way to prevent relapse in schizophrenic patients (33). PP3M is still preferable as it has a longer dosing interval and thus can provide a unique treatment option to help patients achieve improvement in symptoms (52).

Retrospective analyses of population pharmacokinetics of paliperidone using a one-compartment model have shown the apparent clearance (CL), apparent volume of distribution (V), and a fraction of the absorbed dose (F3) to be approximately around 3.84 l/h, 1,960 L and 20.9%, respectively (53). These parameters change accordingly if there is rapid or slow absorption (53). Hence, this study supports the two saturable absorption hypothesis to be applicable for paliperidone after intramuscular administration of its long-acting 3-month formulation, palmitate ester (53). This study was also crucial as it highlighted that factors such as age, race, sex, body mass index, and injection site do not affect the pharmacokinetics and steady-state of paliperidone in patients undergoing such treatment (53). However, the study did show that the renal status of the patient did affect how well the drug was cleared (53). Table 2 summarizes the studies discussed in this section.

**Table 2:** Summary of clinical studies.

**References**	**Phase or purpose**	**Methods**	**Outcome**
Savitz et al. (44)	Phase 3	17-week, flexible-dosed, open-label phase [PP1M: day 1 (150 mg eq. deltoid), day 8 (100 mg eq. deltoid.), weeks 5, 9, and 13 (50, 75, 100, or 150 mg eq., deltoid/gluteal)], clinically stable patients were randomized (1:1) to PP3M (fixed-dose, 175, 263, 350, or 525 mg eq. deltoid/gluteal) or PP1M (fixed-dose, 50, 75, 100, or 150 mg eq. deltoid/gluteal) for a 48-week double-blind phase	PP3M was non-inferior to PP1M: relapse rates were similar in both groups [PP3M: *n* = 37, 8%; PP1M: *n* = 45, 9%; difference in relapse-free rate: 1.2% (95% CI:-2.7%; 5.1%)] No clinically relevant differences were observed in pharmacokinetic exposures between PP3M and PP1M.
Ravenstijn et al. (34)	Phase 1	Multicenter, randomized, open-label, parallel-group study. A total of 328 patients (men or women, aged 18–65 years) were enrolled in 1 of 4 separately conducted panels (A–D). Each panel had 2 single-dose treatment periods [period 1, 1 mg intramuscular paliperidone immediate release (IR); period 2, intramuscular PP3M 75-525 mg eq] separated by a washout of 7–21 days	Peak paliperidone plasma concentration was achieved between 23 and 34 days, and apparent half-life was ~2–4 months Headache and nasopharyngitis were the most common (>7%) treatment-emergent adverse events. Safety and tolerability were similar to those of the 1-month formulation.
Naber et al. (45)	Head-to-head study with aripiprazole	28-week, randomized, non-inferiority, open-label, rater-blinded study between 400 mg of aripiprazole monthly injection (AOM 400) and paliperidone palmitate one monthly injection (PP) Primary endpoint assessed non-inferiority and superiority on QLS total score analyzed using a mixed model for repeated measurements	Statistically significant least squares mean difference in change from baseline to week 28 on QLS total score [4.67 (95% CI: 0.32; 9.02), *p* = 0.036] confirmed non-inferiority and established superiority of AOM 400 vs. PP
Bell Lynum et al. (46)	Comparison of PP3M to placebo to time of relapse	Patients received either PP3M or placebo every 3 months in the double bind phase. The primary efficacy variable was time from randomization to first relapse	A total of 119 patients who entered the double blind phase met the criteria for early illness schizophrenia (PP3M, *n* = 62; placebo, *n* = 57). PP3M significantly delayed time to relapse vs. placebo (*P* = 0.035; hazard ratio, 3.08; 95% CI, 1.08–8.80)
Chirila et al. (47)	Two Phase 3 trials	Occupational status was assessed at each study visit. Logistic regressions modeled the probability of hospitalization during the double-blind phase	At the start of each study, a low percentage of patients were full-time employed or gainfully self-employed (~10% in trial 3012 and 11–13% in trial 3011) Improvement from baseline in occupational status was slightly higher in the PP3M group than in placebo or PP1M groups. Odds of a hospitalization for psychiatric and social reasons during 1 year was 7.74 (95% CI, 2.39–25.05; *p* < 0.001) for a patient on placebo compared with the odds of hospitalization during 1 year for a patient on PP3M. No statistically significant difference was observed between PP3M and PP1M (odds ratio, 1.16; 95% CI, 0.70–1.93).
Savitz et al. (17)	Subanalysis of two phase 3 trials	Patients were randomized to PP3M or PP1M (non-inferiority study A) and PP3M or placebo (study B) in double blind phase. The subgroup analysis included Latin American (Argentina, Brazil, Colombia, Mexico) patients	In study A, relapse-free percentage was similar in Latin America (PP3M: 97%, PP1M: 100%) and rest of world (ROW) (PP3M: 91%, PP1M: 89%). In study B, median time-to-relapse was not estimable in the Latin American subgroup for either placebo or PP3M groups, nor for the ROW PP3M group; the median time-to-relapse in the ROW placebo group was 395 days
Weiden et al. (48)	Examined difference between the three formulations paliperidone	Data were drawn from 3 similarly designed, multicenter, double-blind, placebo-controlled, randomized-withdrawal studies of paliperidone in adults with a schizophrenia diagnosis (according to DSM-IV criteria for ≥1 year before screening): once-daily extended-release oral paliperidone (ORAL paliperidone), once-monthly paliperidone palmitate (PP1M), and once-every-3-months paliperidone palmitate (PP3M).	Postwithdrawal median [95% confidence interval (CI)] days to relapse were 58 days (42–114 days) for ORAL paliperidone, 172 days (134–222 days) for PP1M, and 395 days (274 days-not reached) for PP3M (*P* < 0.0001, pairwise comparisons). Relapse risk was significantly lower (*P* < 0.001) for patients who withdrew from either PP formulation relative to ORAL paliperidone and additionally for patients who withdrew from PP3M relative to PP1M.
Mathews et al. (50)	*Post-hoc*, subgroup analysis	Patients were treated with PP1M [50, 75, 100, or 150 mg equivalent (eq.)] for 17 weeks during an open-label (OL) phase and randomized (1:1) to PP3M (175, 263, 350, or 525 mg eq.) or PP1M (50, 75, 100, or 150 mg eq.) during a 48-week double-blind phase.	Improvements in Positive and Negative Syndrome Scale (PANSS) scores (OL baseline-to-endpoint) were similar in recent-RIS/PALI (oral r isperdal/ paliperidone) [mean (standard deviation):18.3 (17.96)] and no-RIS/PALI [−21.1 (16.40)] subgroups Relapse-free rates were comparable between recent-RIS/PALI [relapse-free rate (95% confidence interval for difference): 2.6 (−4.7 to 10.0); PP3M: 90%; PP1M: 87%] and no-RIS/PALI subgroups [0.8 (−4.5 to 6.0); PP3M: 92%; PP1M: 91%]
Kern Sliwa et al. (51)	Assessment of site pain	Patients (*n* = 1,429) with schizophrenia, treated with PP1M [50–150 mg-eq, 17-week open-label (OL) phase] were randomized to PP1M or PP3M for 48-weeks	Incidence of induration, redness, and swelling were low in both phases (OL: 9–12%; DB: 7–13%), and were mostly mild in both groups
Savitz et al. (52)		Assessed symptomatic and functional remission achieved following paliperidone palmitate 3-month (PP3M) vs. 1-month (PP1M) treatment in patients (age: 18–70 years) with schizophrenia, previously stabilized on PP1M	Functional remission was assessed using Personal and Social Performance scale (PSP). More than 50% patients in both groups achieved symptomatic remission (PP3M: 50.3%; PP1M: 50.8%) during last 6 months of double blind phase. Similar percentage of patients of both groups achieved functional remission (defined as PSP score > 70, PP3M: 42.5%; PP1M: 43.9%) and combined remission (symptomatic and functional remission, PP3M: 25.1%; PP1M: 26.6%) during last 6 months of double blind phase
Magnusson et al. (53)	To characterize the population pharmacokinetics of paliperidone after intramuscular administration of its long-acting 3-month formulation palmitate	Retrospective analysis included pooled data from 651 subjects from one phase I study (single injection of the 3-month formulation) and one phase III study (multiple injections of both 1- and 3-month formulations)	The apparent volume of distribution estimated for the 3-month formulation was not the same as for the previously modeled 1-month formulation Apparent clearance (CL), apparent volume of distribution (V), and fraction of the absorbed dose (F_3_) were estimated to be 3.84 l/h, 1,960 L, and 20.9%

## Conclusion

Schizophrenia is a complex and challenging psychiatric disorder involving a number of dysfunctions with interplaying biological and environmental factors (3, 20). Since its first description in the 19th century, the understanding of schizophrenia has vastly improved and expanded thanks to technological advancements such as gene linkage studies and diagnostic tools such as the DSM (54). Conversely, as research progresses in the classification and treatment of schizophrenia, certain aspects (i.e., treatment options) of the disease remain limited, and more unknowns persist, such as the etiopathogenesis (54).

Pharmacotherapy remains the key treatment for schizophrenia despite only treating the positive and not the negative symptoms (22, 23). However, the use of antipsychotic treatments is necessary to correct the dopamine imbalance, which will yield better results in psychosocial rehabilitation (20). Difficulty in medication management can affect and even derail long-term treatment goals for patients (9, 19). However, PP3M, and potentially the PP6M formulation, offers hope in treatment management. Compared to PP1M, PP3M is just as safe and effective but with the added advantage of increased adherence due to a longer dose interval, decreasing the risk of relapse (19, 44, 48). Despite some safety concerns regarding LAIs and PP3M, the data does not show a significant risk of death in patients taking PP3M or LAIs (38–40). Additionally, many confounding variables contributing to the lowered life spans of individuals with schizophrenia must be taken into account (8). Nevertheless, PP3M is an effective, long-acting treatment option that is enabling patients and providers to focus less on medication adherence and more on the treatment plan and long-term goals (34). Prescribers must consider that not all patients will respond to a LAI and may have to consider other atypical medication trials if the symptoms are not able to be controlled with the PP3M. More research should be done to assess the long term effects of the use of LAIs and to either confirm or refute PP3M, as well as other LAIs, as being a way to prevent disease relapse.

## Author Contributions

All authors listed have made a substantial, direct, and intellectual contribution to the work, and approved it for publication.

## Conflict of Interest

The authors declare that the research was conducted in the absence of any commercial or financial relationships that could be construed as a potential conflict of interest.

## Publisher's Note

All claims expressed in this article are solely those of the authors and do not necessarily represent those of their affiliated organizations, or those of the publisher, the editors and the reviewers. Any product that may be evaluated in this article, or claim that may be made by its manufacturer, is not guaranteed or endorsed by the publisher.
